# A Cluster Randomized Trial of Promoting Alternative Thinking Strategies (PATHS^®^) With Swedish Preschool Children

**DOI:** 10.3389/fpsyg.2021.695288

**Published:** 2021-07-13

**Authors:** Lilianne Eninger, Laura Ferrer-Wreder, Kyle Eichas, Tina M. Olsson, Hanna Ginner Hau, Mara Westling Allodi, Ann-Charlotte Smedler, Mina Sedem, Ingela Clausén Gull, Birgitta Herkner

**Affiliations:** ^1^Department of Psychology, Stockholm University, Stockholm, Sweden; ^2^Department of Psychological Sciences, Tarleton State University, Stephenville, TX, United States; ^3^Department of Social Work, University of Gothenburg, Gothenburg, Sweden; ^4^Department of Special Education, Stockholm University, Stockholm, Sweden

**Keywords:** promoting alternative thinking strategies, cluster randomized controlled trial, social and emotional competence, mental health, preschool, children, universal prevention

## Abstract

The preschool edition of Promoting Alternative THinking Strategies (PATHS^®^) is a school-based, teacher implemented universal intervention developed in the United States designed to promote social emotional competence (SEC) in children as a foundation for improved mental health. PATHS is delivered as a curriculum and it is based on theories and research regarding SEC, brain development, and optimal school environments. A majority of children in Sweden attend preschool, which is government-subsidized and follows a national curriculum focusing on both academic and social emotional learning. However, there is not so much focus on formal instruction nor manual-based lessons. The purpose of this study was to assess the short-term (pre- to post-test) effects of PATHS in the Swedish preschool setting. Using a two-wave cluster randomized trial with multi-method and informant assessment (*N* = 285 4 and 5-year-old Swedish children; *n* = 145 wait-list control; *n* = 140 intervention; *K* = 26 preschools; *k* = 13 intervention; *k* = 13 control) we assessed changes in child emotional knowledge, emotional awareness, social problem solving, prosocial play, inhibitory control, and working memory using structural equation modeling (SEM). We included schools with at least one classroom of 4–5-year-old children from three municipalities. We excluded open preschools, parent cooperative preschools, and family day homes. After random assignment, schools were informed of condition assignment. Research team members were not blind to assignment. We hypothesized that relative to children in control schools, children in intervention schools would evidence improvements in social emotional competence as well as other outcomes. Children in PATHS, relative to children in the control, evidenced improvements in working memory and prosocial play, but also showed an increase in hyperactive behaviors. Girls in PATHS, relative to girls in the control, showed improvement in emotional knowledge and reduced anxiety. These results are considered in light of efforts to promote positive development and mental health. The trial registration number at ClinicalTrials.gov is NCT04512157. Main funding was from Swedish Council for Working Life and Social Research, the Swedish Research Council, Formas, and VINNOVA (dnr: 259-2012-71).

## Introduction

This study concerns the effects of the preschool edition of Promoting Alternative THinking Strategies (PATHS^®^) in a sample of Swedish children and this is the first systematic test of this intervention in Scandinavia. PATHS was originally developed in the United States (U.S.). It is a universal social and emotional learning (SEL) intervention designed to promote social emotional competence (SEC; Domitrovich et al., 2007) which is a protective factor against the later development of mental ill-health including a range of internalizing and externalizing problems (e.g., Durlak et al., 2011). In childhood, SEC involves the integration of cognitive, affective, and behavioral skills and competencies (Bierman et al., 2008b). SEC is instrumental to helping children form and benefit from close relationships, understand emotions, solve problems, and aids them in being able to direct their attention and efforts toward the achievement of their goals (Bierman et al., 2008b).

Social and emotional learning (SEL) represents one of the four main conceptual frameworks instrumental to intervention research that takes a positive rather than solely problematic/prevention-oriented focus and aims (Tolan et al., 2016). SEL intervention approaches have a substantial history as interventions implemented in school settings and are implemented most often with children and adolescents (Tolan et al., 2016). SEL interventions typically have a positive orientation that seek to improve individual functioning and adaption from the starting point of where children currently are in their development. SEL also unites what have historically been separate theories and research fields dedicated to the understanding of the complexity of emotions, executive functioning, and other developmental processes that relate to a wide range of competencies that allow for adaptive functioning in relationships and school (Tolan et al., 2016).

Tolan et al. (2016) in their review of positive development intervention frameworks noted that, SEL as a field, would advance if additional empirical effort was directed at identifying how individual SECs relate to one another across development. In this study, the Collaborative for Academic and Social and Emotional Learning’s (CASEL) five competence domain model (Weissberg et al., 2015; CASEL, 2020) was used as one of the guiding conceptual foundations. In this model, SEC is viewed as a multi-dimensional construct (Weissberg et al., 2015) consisting of self-awareness, self-management, responsible decision making, relationship skills, and social awareness. The CASEL (2020) model provides support for the expectation that growth in child SEC over time is vital to the realization of positive developmental outcomes such as doing adequately to thriving in school academically and socially, to subjectively feeling well, and to being well positioned for the transition to adulthood.

Indeed, there is a growing and clear weight of evidence that the promotion of SECs, for example emotion regulation (e.g., inhibitory control) and cognitive ability (e.g., working memory) are associated with several benefits (e.g., Moltrecht et al., 2020; Sanders et al., 2020). Longitudinal and intervention studies support a prospective association between SEC and mental health. In the U.S. based FAST Track study’s control group, preschool-aged difficulties in SEC significantly predicted outcomes 19 years later in a high-risk subgroup, including greater use of public assistance, police involvement, arrests, severe offenses, binge drinking, marijuana use, medication for social or behavioral issues through high school (Jones et al., 2015). Further, a meta-analysis of 82 school-based universal SEL interventions also indicated several intervention benefits up to a 3-year follow up including significant reductions in conduct problems, emotional distress, and drug use (Taylor et al., 2017). Interventions that promote SEC have substantial potential as a global public health intervention that sets a sound footing for child development and thereby offers a viable strategy to reduce the incidence of mental disorders and other adjustment problems (ISSC, IDS, and UNESCO, 2016; Moltrecht et al., 2020). Although the promise of SEC as a positive development and prevention approach is evident across several studies, more research is needed to identify how the promotion of SEC can be integrated into everyday practice and how to achieve benefits over the long haul in key contexts such as preschools, while considering variation in educational systems across and within nations (e.g., Ferrer-Wreder et al., 2020). Importantly, further insights into the differential effects of interventions to promote SEC in various subgroups is needed as there is evidence that for example girls and boys may differentially benefit from SEL interventions (Bierman et al., 2010).

PATHS is a SEL intervention that fits under the wide conceptual umbrella of positive development interventions (Tolan et al., 2016). Different editions of PATHS exist (e.g., preschool, primary and secondary school). Preschool PATHS is implemented universally in classrooms by trained teachers (Domitrovich et al., 2007) and was designed according to several complementary SEC and educational theories (e.g., emotional intelligence, eco-behavioral systems, the Affective-Behavior-Cognitive-Dynamic Model; Domitrovich et al., 2007). Program modalities and components are guided by a curriculum with 33 interactive lessons involving puppets, stories, role play, and activities such as the use of feeling faces, giving and receiving compliments, use of a self-calming technique, generalization/extension activities, and take-home activities. See the EPISCenter (2011) for the PATHS logic model. Across a school year, PATHS lessons should be implemented weekly and can take place during circle time and last 10–15 min. Teachers are also encouraged to infuse PATHS into their everyday practice as they deem appropriate. Implementation can be encouraged by use of a manualized support model for PATHS teachers (e.g., semi-structured visits to teachers from coaches/supporters during the intervention trial).

Preschool PATHS has been tested in several randomized controlled trials (RCTs) in the U.S. (e.g., Calhoun et al., 2020). Several trials have been conducted through HeadStart child care centers and two of these trials were formative to the design of this study in selection of measures used and analysis strategy. In the first RCT, pre- to post-test intervention-related benefits were found for emotional knowledge (child task), social competence as well as reduced social withdrawal (parent- and/or teacher-rated; Domitrovich et al., 2007; *N* = 246). In a later RCT, PATHS was paired with literacy training (i.e., *REDI*—Research-based, Developmentally Informed; Bierman et al., 2008a,b; *N* = 356). Pre to post-test intervention benefits were found for vocabulary, literacy skills, emotional knowledge, improved social cognitions (child task; Bierman et al., 2008a). Other results also indicated that increases in behavioral executive function (EF) related indicators like walk a line slowly and the child’s task orientation during assessment were important to pre to post-test improvements in print awareness, social competence (teacher- and observer-rated), and reduced aggression (teacher-rated; Bierman et al., 2008b). Five years post-intervention, latent-class growth analysis showed enduring intervention benefits on academic outcomes and children who began behind their peers in EF at the study outset particularly benefited from PATHS in terms of increased EF skills over time (Sasser et al., 2017). A follow up of the REDI cohort, by Bierman et al. (2020), 8–10 years post-intervention (children aged 12–13 and 14–15, respectively) indicated enduring benefits for intervention children, relative to control children in reduced conduct and emotional problems, as well as fewer intervention children evidencing clinically significant problems, namely fewer problems with peers, emotions, and conduct as measured by the Strengths and Difficulties Questionnaire (SDQ). Taken together, the U.S. evidence base on preschool PATHS indicates the intervention has shown an ability to not only protect children in the general population (i.e., universal prevention) but can also be beneficial in protecting higher risk groups of children, for example children exposed to poverty and/or adverse childhood experiences (e.g., better than expected reductions in social-emotional distress and increased school bonding see Gamze et al., 2019; Sanders et al., 2020). Thus, the evidence base for preschool PATHS in the U.S. is generally positive and bodes well for international replication.

Several international effectiveness trials of preschool PATHS have been conducted such as in Croatia (Mihic et al., 2016), Pakistan (Inam et al., 2015), Turkey (Gamze et al., 2019), and the United Kingdom (UK; Berry et al., 2016). These trials demonstrate a broad-based cross-national interest in SEL in young children, feasibility in terms of implementing preschool PATHS in diverse languages, cultures, and educational systems. The aforementioned trials vary in terms of research design as well as in outcome measurement.

Of the non-U.S. preschool PATHS trials, the UK-based trial conducted by Berry et al. (2016) is most similar to the present study. Although the outcome measurement and scale of the trials differ (e.g., the UK trial was much larger than the present study, teacher report only was used in the UK trial), both of the studies used a cluster RCT design, matched analysis to the unit of assignment, and used similar measurement to examine intervention fidelity. In the UK trial, results showed pre- to post-test PATHS related benefits on a teacher-rated scale of SEC, these effects were not present at a follow up, and no changes on mental health as measured by the SDQ were found (post-test nor follow up).

The international effectiveness research literature on preschool PATHS is growing and the present study adds to this literature by reporting on a first of its kind, in Scandinavia, effectiveness trial of preschool PATHS. The Swedish context differs somewhat from the U.S. context in that there is generally less variation in preschool quality as well as household income. In Sweden, most children attend government-subsidized preschools guided by a national curriculum that emphasizes academic learning as well as aspects of social emotional learning. There is typically not so much manualized instruction or formal lessons in preschool, which could make implementation of such a curriculum a challenge. The objective of the study was to investigate, in the Swedish context, the impact of a culturally adapted PATHS intervention on a broad, multimethod spectrum of measures pertaining to SEC. Moderation effects by gender were also of interest to investigate in the Swedish context in particular, due to a clear focus on working toward gender equality in Sweden in school contexts including preschool, as well as due to gender specific effects seen in some U.S. PATHS trials (e.g., Bierman et al., 2010). A cluster RCT was used as the trial design given that PATHS could have school-wide impact, even if only used in part of a school. All study hypotheses and the research question are tested at the cluster level (i.e., at the preschool level). Below, the study hypotheses and research question that guided this study are described.

Hypothesis 1. Relative to children in control schools, children in intervention schools will evidence pre to post-test increases in social emotional competence. Hypothesis 1 breaks down into two sub-hypotheses by measurement and outcome analysis. Because children should evidence normative increases in the targeted primary/secondary outcomes, we expected that PATHS children’s gains would be significantly greater than children in the control condition’s gains on these outcomes.

Hypothesis 1a concerns pre to post-test increases on *primary outcomes* for PATHS children relative to children in the control condition, namely hypothesized intervention-related gains in emotional knowledge/awareness, social problem solving and executive functioning (indexed by inhibitory control and working memory). Hypothesis 1a was measured by child tasks.

Hypothesis 1b involves pre to post-test increases on *secondary outcomes* for PATHS children relative to children in the control group, in terms of hypothesized intervention-related gains in prosocial skills, emotional self-regulation, academic skills, task orientation, social cooperation, social interaction, social independence. Hypothesis 1b was measured with teacher or observer ratings of children.

Hypothesis 2. In comparison to children in control schools, children in PATHS schools will evidence pre to post-test reductions in internalizing behaviors (including social withdrawal and anxiety/somatic problems), externalizing behavior (aggression), as well as significant reductions in inattention and hyperactivity/impulsivity. Hypothesis 2 was measured with teacher ratings of children.

Research Question 1. Given that prior intervention results for the elementary school edition of PATHS indicated moderation of intervention benefits by gender, with unique benefits to boys on reduced ratings of aggression (e.g., Bierman et al., 2010), we explored if girls or boys uniquely benefitted from their participation in preschool PATHS, relative to girls and boys in the control condition.

## Materials and Methods

### Participants

Across two data collection waves, participants were 285 children aged 4–5 years old (*M* = 4.8 years old, *SD* = 6 months; 49% girls) attending 26 preschools (12 intervention, 13 control, 1 school served as control in wave 1 and intervention in wave 2) in three municipalities. Preschools were randomly assigned to intervention (*n* = 145 children; 68 girls, 77 boys) or a wait-list control condition (*n* = 140 children; 73 girls, 67 boys). Data were collected in two waves. Wave 1 began in April 2014 (pre-test) and ended in June 2015 (post-tests). Wave 2 began in April 2015 (pre-test) and ended in June 2016 (post-test).

### Interventions

#### PATHS

Intervention group children (*k* = 14; *n* = 145) participated in the preschool edition of the PATHS curriculum. Teachers participated in a 2-day training led by a certified PATHS trainer. Training involved theoretical bases and empirical evidence of the PATHS program as well as practical examples in lesson coverage. Implementation support was provided monthly to preschool teachers by members of the research group. Approximately 6 months into the program, a 1-day booster session was given by a certified PATHS trainer in order for teachers to receive extra support and as a means of networking and sharing experiences in using the program. Teachers in PATHS classrooms aimed to implement the 33-lesson curriculum. Lessons are interactive and use modeling and support for the use of SEC through the use of children’s literature (i.e., stories), puppets, and role plays as well as activities that help children generalize the lessons outside of time formally dedicated to the intervention (i.e., information to parents, teacher guided extension activities—project and games, emotion coaching during teachable moments). The intervention has a contextual focus and works with teachers to create a classroom climate conducive to the promotion of young children’s SEC by establishing structure and clear expectations in class using positive warm pedagogical techniques. PATHS lessons and extension activities were implemented once a week over the course of a school year (August–May). Lessons took place during group time (i.e., circle time) for about 15–20 min with one extension activity per week (e.g., PATHS game or project). Individual child attendance at PATHS lessons was not monitored; rather, classroom level dosage was estimated using teacher reports of number of lessons implemented.

In 12 out of 14 schools, (86%) there is some type of implementation information in terms of fidelity ratings and/or lesson coverage information. Fidelity ratings ranged from *Neutral* = 3.2 on the modeling subscale to *Does Pretty Well* (a four on a five-point scale) with 3.9 and 3.8 for the teaching and activities subscales, respectively. In a U.S. based preschool PATHS trial, the average fidelity rating across the same subscales/response options was 3.8 (Gamze et al., 2019) and all subscale scores for fidelity average in this trial is 3.6, which is relatively comparable.

#### Wait-List Control

Control group children (*k* = 13; *n* = 140) participated in normal classroom activities during the study.

### Measures

Unless otherwise noted, reliability estimates for scale scores are for children in the present study at pre-test. The measures are described in the order of primary, secondary, and distal outcomes, this distinction was based on the outcome results of two U.S. preschool PATHS trials (Domitrovich et al., 2007; Bierman et al., 2008b) that were formative to the design of the present study. Surveys or child tasks in English were translated into Swedish using a committee approach (Van de Vijver and Leung, 1997). The two translators were both fluent in Swedish and English and well versed in child development and the material to be translated. One translator was a licensed psychologist and other a PhD. One translator did an initial translation that was then reviewed by the second translator and a committee approach procedure was used to resolve any disagreements in word choice so that the meaning in Swedish of any study measure or child task was consistent with the original English edition of the material.

#### Primary Outcome Measures

Child task—Emotional knowledge—The Assessment of Children’s Emotional Skills (ACES; Schultz et al., 2004) measured emotional knowledge. Based on a pilot study, a subsample of photographs (14 from the original 26) were chosen to represent clear facial expressions in children of happiness (4), sadness (2), anger (2), and fear (2), and expressions of mixed emotions (4; fear/anger or sadness/anger). Children were shown each facial expression and asked to respond in terms of what each child in the picture was feeling: happy, sad, angry, scared, or no feeling (varying the order of alternatives for each photo except for no feeling which always came last). One point was given for each correctly identified clear facial expression. The maximum score was 10 (Cronbach’s α = 0.87).

Child task—Emotional Awareness and Social Problem Solving—The Challenging Situations Task (CST; Denham et al., 1994) provides an index of children’s social/interpersonal problem solving. In this study, a modified CST was used consistent with the CST materials and procedure used in the REDI trial (Nix et al., 2013). Four vignettes were presented: (a) a peer knocking down a tower of blocks the child was building; (b) a peer taking the ball the child was playing with; (c) the child being rejected by a peer when he/she asks to play; (d) the child being pushed away by a peer in the queue to the swings. Children were instructed to pretend they were in that situation and asked to say how they would respond. Child open-ended responses were recorded and then coded into the categories: Labels Emotion (emotional awareness), Competent, Aggressive, Inept. Label emotions concerns times when children expressed positive or negative feelings in their responses, such as “I would be mad.” Competent responses were active, non-aggressive responses or attempts to solve the problem, such as “Say stop/no.” Verbally or physically hostile were responses like “Push him/her back.” Inept responses, for example, involved passively ignoring or avoiding the problem such as “Do nothing.” Two researchers coded all CST open-ended responses. Inter-rater reliability was established with Intraclass Correlation Coefficients (ICC) calculated at the scale level with researcher 1 or 2 vs. the ratings of a third researcher who recoded a randomly selected subset of CST data for 27 participants (i.e., approximately 10% of the sample). Each scale score, Labels Emotion (i.e., emotional awareness, ICC = 0.91), Competent (ICC = 0.77), Aggressive (ICC = 0.97), and Inept (ICC = 0.73) represents the sum of each type of response across the four vignettes.

Child task—Inhibitory control 1 (EF1)—The Knock and Tap task is a sub-test of the NEPSY (Korkman et al., 1998) and provides an index of executive functioning (EF), namely inhibition (i.e., within-in task interference control of motor response). The child is instructed to perform specific hand movements in response to the experimenter’s hand movements. For instance, to knock on the table in response to the experimenter placing his/her palm on the table. The total number of trials is 30 and this is the maximum total score, with one point per correct response. The correlation between the first and second part of the task was positive and significant at 0.22, *p* = 0.002.

Child task—Inhibitory control 2 (EF2)—An adapted version of the Day-Night task (Gerstadt et al., 1994) was used to measure inhibition, specifically interference control. Different pictures representing opposites, i.e. up/down and large/small, were presented to children using a computer tablet. For each picture, children were asked to say the opposite word, e.g., to say “down” when presented with an arrow pointing up, thus inhibiting the meaning of the picture shown. The first part of the task consisted of 24 stimuli with the first half being either up/down and the second half large/small. In the second part of the task, which also contained 24 stimuli, the four pictures were presented in a random order. The inter-stimulus-interval was 4,000–4,500 ms, with a presentation time of 1,500 ms in the first part and 1,000 ms in the second part. The maximum score across both trials was 48. Thorell and Wåhlstedt (2006) used the Day-Night task, performed on a computer instead of a computer tablet, with a test-retest reliability of *r* = 0.84.

Child task—Working memory (EF3)—The Word span task is an index of working memory (WM) which is in turn an aspect of EF (Tillman et al., 2008). In the task, a series of words were orally presented to the child, and the child was instructed to remember the words and repeat them in the same order. One and two-syllable words were used. The trials increased from two to six words, with two list series in each trial. The score was calculated as the sum of correct remembered words in the right order, maximum of 30 points (Cronbach’s α = 0.63).

#### Secondary Outcome Measures

Teacher rating—Prosocial/communication skills, emotional self-regulation, academic skills—Social Competence Scale (Sorensen and Dodge, 2016) was rated by children’s teachers who completed items from this entire scale. Items are rated by teachers on a 4-point Likert scale with higher scores indicating better skills. Items are averaged to create subscale scores that included ratings of children’s: Prosocial/communication skills (Cronbach’s α = 0.93, 6 items), emotional self-regulation (Cronbach’s α = 0.94, 9 items), and academic skills (Cronbach’s α = 0.92, 8 items).

Observer rating of child play—Prosocial skills—Social Competence Scale (Sorensen and Dodge, 2016). Seven items from the Prosocial/communication skills subscale of the Social Competence Scale (Sorensen and Dodge, 2016) were used by observers to rate each child’s behavior (i.e., interpersonal relationship skills) during two separate 15-min play sessions. Two raters independently rated each child during the play session in which a total of three children (all study participants) were asked to play with a toy. Play occasion one was with Play Mobile Country Farm and play occasion two was with a Marble Run Play Set. The seven items on this scale were rated on a 5-point Likert scale from 1 (*Not At All*) to 5 (*Very Well*) and there was another option for “did not observe.” The items included behaviors such as “*Shares materials*” and “*is helpful to others.*” Intraclass correlation coefficients for toy 1 and 2 across two raters was 0.93 and 0.92, respectively (two-way random effect, absolute agreement standard) and the correlations between observers’ ratings across the toys were positive and significant (*r* = 0.533 toy 1, *r* = 0.528 toy 2).

Observer rating during child assessment -Task orientation—Task Orientation Scale. A subset of items from a Task Orientation scale, adapted from Smith-Donald et al. (2007) was rated by the child interviewers (research assistants) after children completed all child tasks. This rating reflects the child’s ability to sustain attention across the different assessment tasks. Interviewers rated nine items on a 5-point Likert scale ranging from 0 (*Not True At All*) to 4 (*Very True*) and items included, for example: the child is “*attentive during instructions and demonstrations,” “concentrated,” and “adapts and regulates activity level*” (Cronbach’s α = 0.94).

Teacher rating—social cooperation, interaction, and independence—Preschool and Kindergarten Behavior Scales (PKBS; Merrell, 1996). The PKBS is a teacher-rated survey that is wide ranging and has items/subscales concerning differing children’s social cooperation (Cronbach’s α = 0.90, 11 items), social interaction (Cronbach’s α = 0.89, 10 items), social independence (Cronbach’s α = 0.86, 10 items). Items are rated on 4-point Likert scale from zero to three. Items are averaged into subscale scores with higher values indicating better social skills.

#### Distal Outcome Measures

Teacher rating—internalizing and externalizing behavior—Preschool and Kindergarten Behavior Scales (PKBS; Merrell, 1996). The PKBS is a teacher-rated survey with items/subscales concerning internalizing [e.g., social withdrawal (Cronbach’s α = 0.86, 7 items) and anxiety/somatic symptoms (Cronbach’s α = 0.87, 7 items)] and externalizing behaviors [e.g., aggression (Cronbach’s α = 0.94, 8 items)]. Responses to items are on a 4-point Likert scale from zero to three. Items are averaged into subscale scores with higher values indicating more internalizing or externalizing behaviors.

Teacher rating—Inattention, hyperactivity/impulsivity—ADHD Rating Scale–IV (DuPaul et al., 1998). Items on this teacher-rated scale are designed to provide teachers’ view of inattentive and hyperactive/impulsive behaviors in children. Items are rated on a 4-point Likert scale with the response options 0 (*Never/rarely*), *1 (Sometimes), 2 (Often), and* 3 (*Very Often*) using the time frame of the past 6 months. Subscales used in this study were Inattention (Cronbach’s α = 0.93, 7 items) and Hyperactivity/Impulsivity (Cronbach’s α = 0.93, 9 items).

Implementation Measure and Results—Teachers reported the number of PATHS lessons completed (50% missing data from teachers) and the reported lesson coverage ranged from 0 to 32, the maximum number of PATHS lessons is 33. The average reported lesson coverage was 14.8 lessons (*SD* = 11.7), this amounts to 45% reported lesson coverage.

Members of the research team (called supporters) provided teachers coaching and an occasion to reflect on implementation. During school visits, supporters also rated PATHS teachers on their program fidelity, when observing a PATHS lesson or other classroom activity. Teachers’ fidelity to PATHS was also rated on one occasion by an independent rater. The supporter and independent rater used the same standardized PATHS fidelity rating scale, which has three subscales. Fidelity items were rated on a 5-point scale (1 = *Has Considerable Difficulty*, 2 = *Has Some Difficulty*, 3 = *Neutral*, 4 = *Does Pretty Well*, and 5 = *Highly Skilled*).

In terms of intervention fidelity ratings, the Modeling subscale consists of five items (e.g., *“It is clear when you enter this classroom and look around that it is a PATHS classroom” M* = 3.2, *SD* = 0.80, 29% missing data, Cronbach’s α = 0.95, ICC supporter vs. rater = 0.62). The Teaching subscale has five items (e.g., *“Teacher is generally prepared for PATHS activities and seems familiar with what to do”*; *M* = 3.9, *SD* = 0.58, 29% missing data, Cronbach’s α = 0.82, ICC supporter vs. rater = 0.86). The Activities subscale had four items (e.g., *“building a caring classroom: Class structure”*; *M* = 3.8, *SD* = 0.67, 36% missing data, Cronbach’s α = 0.90, ICC supporter vs. rater = 0.73).

### Procedure

Prior to the intervention trial, a series of formative research studies were conducted over a 2-year period in order to culturally adapt PATHS to an urban multi-cultural Swedish preschool context. See Ferrer-Wreder et al., 2021 for a description of the cultural adaptation process. Once the formative research was completed, the intervention trial began. Schools in three municipalities in a large Swedish city took part in this study. These municipalities were selected because they represented a wide range of community types with respect to average income and other socioeconomic factors. Educational administrations at the municipal level provided assent to recruit schools and then recruitment shifted to school principals and teachers with pupils who were 4–5 years old, and it was required that principals and teachers of 4–5-year-old children agreed to participate in the trial.

After random assignment of schools to study condition (see Supplementary Appendix 1B for randomization procedure details), intervention teachers took part in a 2-day training by a certified PATHS trainer as well as a 1-day booster training. Pre- and post-test assessments were carried similarly, with pre-test assessments being made at the beginning of the school year (and before the training in intervention schools) and post-test assessments conducted toward the end of the school year. The child tasks were administered by two trained research assistants with participating children individually during visits to all participating preschools (intervention and wait-list control). The tasks were distributed in a fixed order across two sessions lasting approximately 30 min each, on the same day or spread across two different days. Teachers were also asked to provide ratings related to participating children and received movie vouchers as compensation, and each participating classroom received a $55 bookstore gift card. This study was approved by a regional ethics review panel (dnr. 2012/1714-31/5). Parents provided written consent for child participation and children provided verbal assent regarding their study participation. All authors have no known conflicts of interest and certify their responsibility for this article. The protocol was registered at ClinicalTrials.gov (NCT04512157) after the trial was completed.

### Data Analysis Strategy

We evaluated the pre to post-test effects of preschool PATHS by specifying outcome models for each of the outcome variables and used them to examine covariate-adjusted change (Rausch et al., 2003). In each of the models, PATHS was a dichotomous variable (1 = intervention, 0 = comparison group) hypothesized to predict post-test scores on indicators among the primary, secondary, and distal outcomes, holding constant the pre-test scores of the same indicator. Male (1 = male, 0 = female), age (in months), and Community were included as covariates of the outcome variables. Schools were located in three municipalities (i.e., communities), therefore the community variable was represented by a pair of dichotomous variables: Community 2 (1 = Community 2, 0 = Community 1) and Community 3 (1 = Community 3, 0 = Community 1).

Each model was just-identified. Therefore, no model fit indices were generated. We explored moderation of intervention effects by gender by adding an interaction term (PATHS ^∗^ Male; Jaccard and Turrisi, 2003) as a predictor of each outcome. Statistically non-significant interaction paths were then trimmed from the models. We generated the parameter estimates using structural equation modeling (SEM) in Mplus 8.0 (Muthén and Muthén, 1998–2017) with a robust maximum likelihood estimator and a Huber-White adjustment to the standard errors to account for nesting of participants within schools. With respect to power, for a contrast of means between two independent groups and an effect size corresponding to Cohen’s (1988) definition of a medium effect—that is, a standardized path coefficient of0.30 (Durlak, 2009)—the sample size needed to achieve power of0.80 is 64 per group (Cohen, 1988). The sample size meets this criterion for both the comparison of the PATHS and control conditions and comparisons of boys and girls within the PATHS and control conditions.

We used multiple imputation to cope with missing data. Specifically, we used a Bayesian procedure in Mplus in which imputed data are generated through a Markov Chain Monte Carlo simulation (Asparouhov and Muthén, 2010). In this procedure, imputed data are generated by sampling from the posterior distribution of a missing data model. The process is repeated many times to create multiple imputed datasets that can then be analyzed using other estimators (e.g., robust maximum likelihood). We used an unrestricted H1 variance-covariance model to impute 50 datasets. We specified two Markov Chain Monte Carlo chains, the Gibbs sampler, a thinning interval of 500, and a convergence criterion of0.05. The reported results were pooled from the 50 imputed datasets.

## Results

The missing data rate for single items ranged from 9 to 33% at pre-test and from 13 to 38% at post-test. Table 1 provides the unadjusted means and standard deviations by condition across pre and post-tests for all outcomes. Parameter estimates, with no covariates in the models are shown in Table 2. Parameter estimates with covariates in the models (age, community, gender) are shown in Table 3. The estimated path coefficient is the mean difference between the intervention and control groups in the post-test score. This provides an estimate of intervention effect. The standardized path estimates presented in Tables 2, 3 are standardized with respect to the outcome variable but not the condition variable, providing a standardized effect size estimate. Specifically, these standardized path coefficients are equal to the mean difference between the groups, holding constant the other predictors (i.e., the path coefficient), divided by the standard deviation of the outcome variable.

**TABLE 1:** Unadjusted means and standard deviations, *N* = 285.

	Intervention (*n* = 145)				Control (*n* = 140)			
	Pre-test		Post-test		Pre-test		Post-test	
	*M*	*SD*	*M*	*SD*	*M*	*SD*	*M*	*SD*
**Primary outcomes (CT)**								
ACES-emotional know.	6.987	1.883	7.756	1.356	6.995	1.642	7.452	1.439
CST-emotional awareness	4.028	1.526	4.404	1.322	4.372	1.841	4.385	1.386
CST-SPS: competent	2.410	2.337	3.046	2.346	2.383	2.148	2.762	2.179
CST-SPS: aggressive	0.940	1.571	0.509	1.150	0.589	1.156	0.670	1.561
CST-SPS: inept	0.622	1.176	0.570	1.110	0.522	1.027	0.501	1.003
IC1: knock and Tap	23.817	6.534	25.800	5.669	23.456	6.498	25.033	5.620
IC2: day-night task	26.578	13.988	31.912	13.233	27.091	15.004	34.680	11.586
WM: word span task	10.769	4.553	13.250	4.421	10.850	4.509	11.662	4.516
**Secondary outcomes**								
Prosocial/communication skills (T)	2.918	0.889	3.035	0.908	2.876	0.880	3.152	0.837
Emotional self-regulation (T)	2.716	0.933	2.704	1.017	2.663	0.919	2.800	0.970
Academic skills (T)	3.104	0.887	3.188	1.006	3.081	0.852	3.293	1.002
Prosocial skills (O)	3.594	0.742	3.783	0.737	3.645	0.725	3.599	0.611
Task orientation (O)	2.901	0.850	3.019	0.874	2.923	0.910	3.008	0.838
Social cooperation (T)	2.632	0.447	2.593	0.514	2.610	0.430	2.683	0.458
Social interaction (T)	2.311	0.425	2.463	0.513	2.465	0.516	2.628	0.470
Social independence (T)	2.642	0.425	2.695	0.436	2.685	0.399	2.774	0.349
**Distal outcomes**								
Social withdrawal (T)	0.506	0.576	0.525	0.631	0.734	0.595	0.654	0.623
Anxiety/somatic symptoms (T)	0.370	0.517	0.397	0.546	0.549	0.581	0.577	0.627
Aggression (T)	0.422	0.699	0.572	0.782	0.453	0.612	0.452	0.720
Inattention (T)	0.782	0.774	0.960	0.763	0.674	0.780	0.519	0.754
Hyperactivity/impulsivity (T)	0.685	0.706	0.857	0.871	0.625	0.740	0.552	0.787

*CT, Child task; Emotional Know., Emotional Knowledge; CST, The Challenging Situations Task; SPS, Social problem solving; IC1, Inhibitory control 1; IC2, Inhibitory control 2; WM, Working Memory; T, Teacher rated index, O, Observer rated index.*

**TABLE 2:** Parameter estimates, *N* = 285.

	Estimate	*p*	95% CI	St. Est.	95% CI
**Primary outcomes**					
ACES-emotional knowledge	0.305	0.102	[−0.061, 0.670]	0.217	[−0.040, 0.474]
CST-emotional awareness	0.051	0.792	[−0.329, 0.432]	0.038	[−0.241, 0.316]
CST-SPS: competent	0.276	0.473	[−0.477, 1.029]	0.121	[−0.209, 0.451]
CST-SPS: aggressive	–0.239	0.154	[−0.567, 0.090]	–0.174	[−0.399, 0.051]
CST-SPS: inept	0.066	0.685	[−0.254, 0.387]	0.062	[−0.238, 0.363]
IC1: knock and Tap task	0.651	0.348	[−0.709, 2.011]	0.115	[−0.117, 0.347]
IC2: day-night task	–2.522	0.091	[−5.443, 0.399]	–0.201	[−0.431, 0.029]
WM: word span task	1.629	0.005	[0.493, 2.765]	0.359	[0.120, 0.597]
**Secondary outcomes**					
Prosocial/communication skills	–0.143	0.305	[−0.415, 0.130]	–0.162	[−0.469, 0.144]
Emotional self-regulation	–0.135	0.249	[−0.365, 0.094]	–0.135	[−0.363, 0.093]
Academic skills	–0.119	0.431	[−0.416, 0.178]	–0.118	[−0.205, 0.087]
Prosocial skills (observer)	0.198	0.119	[−0.051, 0.448]	0.290	[−0.074, 0.655]
Task orientation	0.022	0.880	[−0.269, 0.314]	0.026	[−0.314, 0.367]
Social cooperation	–0.107	0.091	[−0.230, 0.017]	–0.217	[−0.471, 0.036]
Social interaction	–0.091	0.209	[−0.231, 0.051]	–0.183	[−0.470, 0.105]
Social independence	–0.056	0.287	[−0.159, 0.047]	–0.140	[−0.392, 0.112]
**Distal outcomes**					
Social withdrawal	0.029	0.721	[−0.129, 0.187]	0.046	[−0.205, 0.297]
Anxiety/Somatic symptoms	–0.068	0.389	[−0.222, 0.086]	–0.115	[−0.373, 0.144]
Aggression	0.143	0.133	[−0.043, 0.330]	0.189	[−0.058, 0.436]
Inattention	0.166	0.152	[−0.061, 0.393]	0.215	[−0.083, 0.513]
Hyperactivity/Impulsivity	0.250	0.009	[0.062, 0.438]	0.295	[0.077, 0.514]

**TABLE 3:** Parameter estimates, covariate adjusted models; *N* = 285.

	Estimate	*p*	95% CI	St. Est.	95% CI
**Primary outcomes**					
ACES-emotional knowledge	0.318	0.059	[−0.012, 0.648]	0.226	[−0.006, 0.458]
CST-emotional awareness	–0.004	0.983	[−0.394, 0.386]	–0.003	[−0.291, 0.285]
CST-competent	0.409	0.218	[−0.242, 1.059]	0.180	[−0.104, 0.464]
CST-aggressive	–0.242	0.126	[−0.551, 0.068]	–0.176	[−0.389, 0.037]
CST-inept	0.074	0.601	[−0.204, 0.353]	0.070	[−0.191, 0.332]
Knock and tap task	0.882	0.181	[−0.411, 2.175]	0.156	[−0.062, 0.374]
Day-night task	–1.637	0.281	[−5.551, 1.341]	–0.130	[−0.367, 0.106]
Word span task	1.760	0.002	[0.627, 2.894]	0.387	[0.153, 0.622]
**Secondary outcomes**					
Prosocial/Communication	–0.150	0.263	[−0.414, 0.113]	–0.172	[−0.468, 0.125]
Emotional self-regulation	–0.153	0.188	[−0.380, 0.075]	–0.152	[−0.377, 0.072]
Academic skills	–0.113	0.468	[−0.418, 0.192]	–0.112	[−0.413, 0.189]
Prosocial skills (observer)	0.277	0.010	[0.065, 0.490]	0.406	[0.093, 0.719]
Task orientation	0.073	0.616	[−0.213, 0.360]	0.086	[−0.250, 0.422]
Social cooperation	–0.093	0.121	[−0.210, 0.025]	–0.189	[−0.431, 0.053]
Social interaction	–0.082	0.245	[−0.221, 0.057]	–0.164	[−0.443, 0.114]
Social independence	–0.052	0.314	[−0.153, 0.049]	–0.130	[−0.378, 0.118]
**Distal outcomes**					
Social withdrawal	0.040	0.626	[−0.122, 0.202]	0.064	[−0.194, 0.322]
Anxiety/Somatic symptoms	–0.080	0.289	[−0.228, 0.068]	–0.135	[−0.382, 0.112]
Aggression	0.130	0.139	[−0.042, 0.303]	0.172	[−0.056, 0.401]
Inattention	0.129	0.260	[−0.096, 0.355]	0.168	[−0.126, 0.462]
Hyperactivity/Impulsiveness	0.208	0.031	[0.019, 0.397]	0.245	[0.024, 0.467]

Table 3 shows the differences in outcome scores at post-test, holding constant the pre-test score and a set of covariates added to outcome models (age, gender, community). The choice of covariates is consistent with other preschool PATHS trials. Because of space limitations, the description of the results described focuses on those changes in the primary, secondary, and distal outcomes that evidenced a group difference (intervention relative to comparison schools) from pre to post-test with a standardized effect size estimate of ± 0.20 or higher across both the non-covariate adjusted and covariate adjusted analyses. Although this effect size is generally considered to reflect a small effect (Cohen, 1988), it is consistent with initial benchmarks developed through meta-analyses of SEL interventions (Taylor et al., 2017) and positive development interventions (Ciocanel et al., 2017), as well as earlier findings that measures of behavior yield relatively small effect sizes in psychosocial interventions for youth (Durlak, 2009). Specifically, Taylor et al. (2017) identified SEL intervention post-test effect sizes of 0.17 for SEL skills and between 0.06 and 0.22 for behavioral, emotional, and academic outcomes. Similarly, Ciocanel et al. (2017) identified positive development intervention effect sizes between 0.04 and 0.22 for behavioral, emotional, and academic outcomes. A more detailed description of Tables 2, 3 results is provided in Supplementary Appendix 1.

### Primary Outcomes

Primary outcomes examining hypothesis 1a included eight scores indexing emotional knowledge and awareness, social problem solving, executive functioning—inhibitory control and working memory. Out of the eight scores, two scores had effect sizes ± 0.20 and higher in the non-covariate and covariate adjusted analyses. The difference in the emotion knowledge score at post-test between the intervention and control group was estimated to be 0.31 [−0.06,0.67]. The effect size, as indexed by the standardized path coefficient was 0.22 [−0.04,0.47] (see Table 2). As shown in Table 3, the difference in the emotional knowledge score at post-test between the intervention and control group was estimated to be 0.32 [−0.01,0.65], holding constant age, gender, and community. The standardized path coefficient was 0.23 [−0.01,0.46]. In sum, effect sizes ranged from 0.22 to 0.23 across analyses for emotional knowledge, and the intervention-related change was as hypothesized. In other words, children in PATHS schools showed greater gains in emotional knowledge from pre to post-test relative to children in the wait-list control schools (see Table 1).

As shown in Table 2, the difference between groups on post-test working memory was estimated to be 1.63 [0.49, 2.77], with a standardized coefficient of 0.36 [0.12,0.60]. In the covariate adjusted analyses (Table 3), the difference between groups on post-test working memory was estimated to be 1.76 [0.63, 2.89] holding constant the pre-test score, age, gender, and community, with a standardized coefficient of 0.39 [0.15,0.62]. The effect sizes for change in working memory ranged from 0.36 to 0.39 across analyses and the direction of the change was as hypothesized, with greater gains in working memory for PATHS children relative to children in wait-list control schools (see Table 1).

Out of the eight scores for primary outcomes, five scores, namely CST—emotional awareness, CST—competent, CST—aggressive, CST—inept, inhibitory control 1 (Knock and Tap test), evidenced standardized coefficients less than ± 0.20 across non-adjusted and covariate adjusted analyses. One out of the eight primary outcomes, inhibitory control 2 (Day-Night task) showed a standardized coefficient of −0.20 in the analyses with no covariates, however, the effect size decreased in the covariate adjusted analyses to −0.13.

### Secondary Outcomes

Secondary outcomes examining hypothesis 1b included eight scores indexing key outcomes such as prosocial skills, emotional self-regulation, academic skills, task orientation, social cooperation, interaction, and independence. Out of the eight scores, one score had an effect size ± 0.20 and higher in both the non-adjusted and covariate adjusted analyses. As seen in Table 2, the estimated difference between study conditions in observer-rated prosocial skills across two play observations was 0.20 [−0.05,0.45], with a standardized coefficient of 0.29 [−0.07,0.66]. As shown in Table 3, the estimated difference between the groups in observer-rated prosocial skills as measured in the play observation was 0.28 [0.07,0.49], with a standardized coefficient of 0.41 [0.09,0.72]. In sum, for the play observations, effect sizes ranged from 0.29 to 0.41 and intervention-related change was as hypothesized, with PATHS children having evidenced an increase in observer-rated prosocial skills from pre to post-test relative to children in wait-list control schools (see Table 1).

Out of the eight scores for secondary outcomes, six scores namely teacher-rated prosocial/communication skills, emotional self-regulation, academic skills, social interaction and independence, as well as observer-rated task orientation during the child assessment battery showed standardized coefficients less than ± 0.20 across non-covariate and covariate adjusted analyses. One out of the eight secondary outcomes, teacher-rated social cooperation showed a standardized coefficient of −0.22 in analyses with no covariates, but the effect size decreased in the covariate adjusted analyses to −0.19.

### Distal Outcomes

Distal outcomes tested in hypothesis 2 consisted of five scores measuring teacher-rated child internalizing and externalizing behavior as well as inattention and hyperactivity/impulsivity. Out of the five scores, one score had an effect size ± 0.20 and higher in both the non-adjusted and covariate adjusted analyses. As seen in Table 2, the estimated group difference was 0.25 [0.06, 0.44] for hyperactivity/impulsivity with a standardized estimate of 0.30 [0.08, 0.51]. In Table 3, the estimated group difference in the covariate adjusted analyses was 0.21 [0.02, 0.40] for hyperactivity/impulsivity, with a standardized estimate of 0.25 [0.02, 0.47]. In sum, for these teacher ratings, effect sizes ranged from 0.30 to 0.25 and intervention-related change was not in the hypothesized direction, with PATHS children having evidenced an increase in teacher-rated hyperactivity/impulsivity from pre to post-test relative to children in wait-list control schools (see Table 1). The unadjusted means and standard deviations on this score for PATHS children was *M* = 0.69 (*SD* = 0.71) at pre-test and *M* = 0.86 (*SD* = 0.87) relative to control children which was *M* = 0.63 (*SD* = 0.74) at pre-test and *M* = 0.55 (*SD* = 0.79). Teacher-rated hyperactivity/impulsivity items are rated by teachers on a four-point scale (0 = *Never/Rarely*, 1 = *Sometimes*, 2 = *Often*, and 3 = *Very Often*). Out of the five scores for distal outcomes, three scores namely teacher-rated social withdrawal, anxiety/somatic symptoms and aggression evidenced standardized coefficients less than ± 0.20 across non-covariate and covariate adjusted analyses. One out of the five distal outcomes, teacher-rated inattention showed a standardized coefficient of 0.22 in analyses with no covariates, but the effect size decreased in the covariate adjusted analyses to 0.17.

### Intervention Moderation Analysis

In order to explore research question 1, we evaluated potential moderation of intervention effects according to gender by adding interaction terms (PATHS ^∗^ Female; Jaccard and Turrisi, 2003) to the covariate models as predictors of each outcome (primary, secondary, and distal outcomes). Statistically non-significant interaction paths were then trimmed from the models. Two plausible interaction effects were found. First, there was a PATHS ^∗^ Female interaction effect on emotional knowledge, path = −0.91, [0.13, 1.69], *p* = 0.02. Among girls, the difference between the intervention and control groups was 0.78, [0.20, 1.35], with a standardized path coefficient of 0.55 [0.15, 0.95]. This estimate for boys was negative, −0.13, [−0.58, 0.31], a standardized coefficient of −0.10 [−0.41, 0.22]. Examination of the subgroup means and standard deviations (see Table 4) revealed that girls in PATHS increased in emotional knowledge from pre-test (*M* = 7.03, *SD* = 1.71) to post-test (*M* = 8.01, *SD* = 1.25). Girls in the control condition showed lesser increases from pre-test (*M* = 7.12, *SD* = 1.60) to post-test (*M* = 7.25, *SD* = 1.51). Boys in PATHS also increased from pre-test (*M* = 6.95, *SD* = 2.02) to post-test (*M* = 7.53, *SD* = 1.41). However, boys in the control condition increased slightly more from pre-test (*M* = 6.87, *SD* = 1.67) to post-test (*M* = 7.67, *SD* = 1.33).

**TABLE 4:** Unadjusted means and standard deviations, boys, and girls, *N* = 285.

		Intervention (*n* = 145)				Control (*n* = 140)			
		Pre-test		Post-test		Pre-test		Post-test	
		*M*	*SD*	*M*	*SD*	*M*	*SD*	*M*	*SD*
**Primary outcomes (CT)**									
ACES-emotional knowledge	Boys	6.947	2.023	7.528	1.405	6.965	1.672	7.667	1.324
	Girls	7.032	1.707	8.013	1.246	7.115	1.603	7.254	1.510
CST-emotional awareness	Boys	3.961	1.453	4.333	1.301	4.354	1.849	4.284	1.338
	Girls	4.103	1.600	4.485	1.341	4.389	1.832	4.476	1.422
CST-SPS: competent	Boys	2.300	2.293	2.886	2.253	2.581	2.134	2.361	2.037
	Girls	2.533	2.378	3.226	2.432	2.201	2.144	3.130	2.238
CST-SPS: aggressive	Boys	1.274	1.866	0.749	1.344	0.818	1.410	0.818	1.858
	Girls	0.562	1.021	0.237	0.796	0.379	0.802	0.533	1.211
CST-SPS: inept	Boys	0.604	1.040	0.634	1.164	0.768	1.246	0.604	1.123
	Girls	0.642	1.311	0.498	1.038	0.297	0.700	0.407	0.868
IC1: knock and Tap	Boys	23.387	6.310	25.674	5.592	22.934	7.186	24.313	6.381
	Girls	24.305	6.740	25.943	5.743	23.936	5.749	25.693	4.715
IC2: day-Night task	Boys	24.695	14.687	29.955	13.654	27.383	14.871	33.527	11.364
	Girls	28.711	12.802	34.128	12.358	26.823	15.113	35.739	11.681
WM: word span task	Boys	10.643	4.487	12.938	4.555	10.759	4.217	12.169	4.732
	Girls	10.913	4.620	13.128	4.253	10.933	4.758	11.197	4.254
**Secondary outcomes**									
Prosocial/communication skills	Boys	2.749	0.962	2.811	0.978	2.655	0.893	3.009	0.893
	Girls	3.109	0.753	3.289	0.743	3.078	0.816	3.282	0.758
Emotional self-regulation	Boys	2.529	1.024	2.403	1.089	2.532	0.917	2.643	0.985
	Girls	2.927	0.763	3.046	0.801	2.783	0.903	2.943	0.932
Academic skills	Boys	2.837	0.961	2.866	1.067	2.872	0.848	3.163	1.033
	Girls	3.406	0.675	3.552	0.783	3.273	0.808	3.413	0.955
Prosocial skills (observer)	Boys	3.536	0.780	3.633	0.720	3.505	0.835	3.506	0.659
	Girls	3.660	0.690	3.952	0.718	3.774	0.578	3.684	0.549
Task orientation	Boys	2.787	0.780	2.872	0.858	2.803	0.975	2.945	0.921
	Girls	3.030	0.856	3.187	0.860	3.034	0.825	3.066	0.749
Social cooperation	Boys	2.494	0.505	2.434	0.572	2.545	0.421	2.619	0.473
	Girls	2.788	0.319	2.773	0.362	2.670	0.430	2.742	0.436
Social interaction	Boys	2.165	0.620	2.298	0.550	2.316	0.528	2.497	0.501
	Girls	2.478	0.503	2.649	0.392	2.601	0.463	2.748	0.402
Social independence	Boys	2.554	0.465	2.622	0.499	2.579	0.443	2.708	0.392
	Girls	2.743	0.349	2.777	0.330	2.783	0.322	2.834	0.290
**Distal outcomes**									
Social withdrawal	Boys	0.637	0.627	0.635	0.699	0.869	0.630	0.716	0.620
	Girls	0.356	0.471	0.401	0.514	0.611	0.531	0.597	0.619
Anxiety/somatic symptoms	Boys	0.408	0.540	0.511	0.597	0.642	0.647	0.600	0.616
	Girls	0.327	0.484	0.268	0.446	0.463	0.495	0.557	0.635
Aggression	Boys	0.596	0.787	0.798	0.844	0.624	0.674	0.619	0.757
	Girls	0.225	0.517	0.316	0.611	0.295	0.499	0.298	0.646
Inattention	Boys	1.004	0.848	1.020	0.788	0.762	0.809	0.619	0.754
	Girls	0.529	0.585	0.455	0.605	0.593	0.743	0.427	0.741
Hyperactivity/impulsivity	Boys	0.833	0.719	1.147	0.914	0.700	0.811	0.700	0.841
	Girls	0.517	0.653	0.528	0.684	0.557	0.660	0.416	0.706

Second, there was also a PATHS ^∗^Female interaction effect on anxiety/somatic symptoms, path = −0.25, [−0.49, −0.01], *p* = 0.04. Among girls, the difference between the intervention and control groups was −0.20, [−0.40, −0.01], with a standardized path coefficient of −0.35 [−0.67, −0.02]. This estimate for boys was 0.05, [−0.14, 0.23], with a standardized coefficient of 0.08 [−0.23, 0.38]. Examination of the subgroup means and standard deviations revealed that girls in PATHS decreased in anxiety/somatic symptoms from pre-test (*M* = 0.33, *SD* = 0.48) to post-test (*M* = 0.27, *SD* = 0.45), while girls in the control condition increased from pre-test (*M* = 0.46, *SD* = 0.50) to post-test (*M* = 0.56, *SD* = 0.82). Conversely boys in PATHS increased from pre-test (*M* = 0.41, *SD* = 0.54) to post-test (*M* = 0.51, *SD* = 0.60), while boys in the control condition decreased from pre-test (*M* = 0.64, *SD* = 0.65) to post-test (*M* = 0.60, *SD* = 0.62). Results from all tested moderation models are presented in Table 5.

**TABLE 5:** Interaction parameter estimates, interaction models, *N* = 285.

Outcomes	Predictors	Estimate	*p*	95% CI	St. Est
**Primary outcomes**					
ACES-emotional knowledge	PATHS*Gender	0.911	0.022	[0.134, 1.688]	0.648
	PATHS-Female	0.777	0.008	[0.204, 1.350]	0.553
	PATHS-Male	–0.134	0.550	[−0.575, 0.306]	–0.096
CST-emotional awareness	PATHS*Gender	–0.051	0.861	[−0.627, 0.524]	–0.038
	PATHS-Female	–0.030	0.892	[−0.463, 0.403]	–0.022
	PATHS-Male	0.021	0.937	[−0.508, 0.551]	0.016
CST-SPS: competent	PATHS*Gender	–0.581	0.247	[−1.566, 0.404]	–0.256
	PATHS-Female	0.116	0.780	[−0.696, 0.927]	0.051
	PATHS-Male	0.697	0.088	[−0.103, 1.497]	0.307
CST-SPS: aggressive	PATHS*Gender	–0.116	0.442	[−0.410, 0.179]	–0.153
	PATHS-Female	0.072	0.528	[−0.152, 0.296]	0.095
	PATHS-Male	0.188	0.108	[−0.041, 0.416]	0.248
CST-SPS: inept	PATHS*Gender	0.080	0.716	[−0.351, 0.511]	0.076
	PATHS-Female	0.115	0.511	[−0.228, 0.457]	0.108
	PATHS-Male	0.035	0.851	[−0.328, 0.397]	0.033
IC1: knock and tap task	PATHS*Gender	–1.097	0.383	[−3.560, 1.366]	–0.194
	PATHS-Female	0.330	0.671	[−1.193, 1.854]	0.059
	PATHS-Male	1.427	0.164	[−0.582, 3.437]	0.252
IC2: day-Night task	PATHS*Gender	–0.154	0.950	[−4.995, 4.687]	–0.013
	PATHS-Female	–1.715	0.405	[−5.755, 2.325]	–0.137
	PATHS-Male	–1.562	0.400	[−5.195, 2.072]	–0.124
WM: word span task	PATHS*Gender	1.573	0.088	[−0.233, 3.379]	0.347
	PATHS-Female	2.552	< 0.001	[1.116, 3.989]	0.562
	PATHS-Male	0.979	0.183	[−0.463, 2.421]	0.215
**Secondary outcomes**					
Prosocial/Communication skills	PATHS*Gender	0.255	0.190	[−0.126, 0.636]	0.291
	PATHS-Female	–0.022	0.893	[−0.350, 0.305]	–0.025
	PATHS-Male	–0.277	0.086	[−0.594, 0.039]	–0.316
Emotional self-regulation	PATHS*Gender	0.231	0.187	[−0.112, 0.573]	0.232
	PATHS-Female	–0.036	0.801	[−0.316, 0.244]	–0.035
	PATHS-Male	–0.267	0.069	[−0.554, 0.021]	–0.267
Academic skills	PATHS*Gender	0.339	0.137	[−0.108, 0.786]	0.337
	PATHS-Female	0.058	0.759	[−0.315, 0.432]	0.059
	PATHS-Male	0.339	0.140	[−0.108, 0.786]	–0.278
Prosocial skills (observer)	PATHS*Gender	0.147	0.260	[−0.108, 0.401]	0.214
	PATHS-Female	0.257	0.006	[0.099, 0.603]	0.514
	PATHS-Male	0.205	0.093	[−0.034, 0.444]	0.300
Task orientation	PATHS*Gender	0.174	0.335	[−0.180, 0.528]	0.203
	PATHS-Female	0.161	0.288	[−0.136, 0.458]	0.188
	PATHS-Male	–0.013	0.943	[−0.378, 0.351]	–0.015
Social cooperation	PATHS*Gender	0.091	0.312	[−0.085, 0.266]	0.185
	PATHS-Female	–0.047	0.541	[−0.197, 0.104]	–0.095
	PATHS-Male	–0.137	0.056	[−0.279, 0.004]	–0.280
Social interaction	PATHS*Gender	0.087	0.463	[−0.146, 0.321]	0.176
	PATHS-Female	–0.038	0.633	[−0.196, 0.119]	–0.076
	PATHS-Male	–0.126	0.223	[−0.328, 0.077]	–0.252
Social independence	PATHS*Gender	0.038	0.651	[−0.128, 0.204]	0.097
	PATHS-Female	–0.033	0.622	[−0.163, 0.097]	–0.081
	PATHS-Male	–0.071	0.290	[−0.202, 0.060]	–0.179
**Distal outcomes**					
Social withdrawal	PATHS*Gender	–0.097	0.428	[− 0.337, 0.143]	–0.154
	PATHS-Female	–0.008	0.935	[−0.210, 0.193]	–0.013
	PATHS-Male	0.089	0.395	[−0.115, 0.292]	0.141
Anxiety/Somatic symptoms	PATHS*Gender	–0.249	0.041	[−0.489, -0.010]	–0.420
	PATHS-Female	–0.204	0.044	[−0.403, -0.006]	–0.345
	PATHS-Male	0.045	0.628	[−0.137, 0.227]	0.076
Aggression	PATHS*Gender	–0.116	0.442	[−0.410, 0.179]	–0.153
	PATHS-Female	0.072	0.528	[−0.152, 0.296]	0.095
	PATHS-Male	0.188	0.108	[−0.041, 0.416]	0.248
Inattention	PATHS*Gender	–0.185	0.196	[−0.465, 0.095]	–0.241
	PATHS-Female	0.037	0.805	[−0.255, 0.328]	0.047
	PATHS-Male	0.221	0.058	[−0.007, 0.450]	0.288
Hyperactivity/Impulsivity	PATHS*Gender	–0.171	0.139	[−0.398, 0.056]	–0.203
	PATHS-Female	0.122	0.237	[−0.080, 0.323]	0.143
	PATHS-Male	0.293	0.012	[0.064, 0.522]	0.346

*^∗^interaction model.*

## Discussion

This study contributes to the field of imported interventions by investigating the effectiveness of the culturally adapted preschool PATHS program in a Swedish context. The trial included a broad spectrum of SEC measures, and employed multiple methods. We hypothesized that children in the intervention group compared to the control group would show larger gains in social emotional competence (both primary and secondary outcomes) as well as reductions in internalizing and externalizing behaviors (distal outcomes). We found support for intervention-related increases in emotional knowledge, working memory and prosocial play (representing several domains of SEC). With respect to distal outcomes, there was some support for intervention-related reductions in internalizing behavior, specifically anxiety/somatic symptoms, although this was specific for girls. Somewhat surprisingly, there was an increase in hyperactivity/impulsivity related to the intervention.

Consistent with prior U.S. based preschool PATHS intervention trials (e.g., Domitrovich et al., 2007; Bierman et al., 2008b), this study thus showed clear evidence for some, but not all, of the hypothesized pre- to post-test benefits that have already been associated with the use of preschool PATHS. Although only three scale scores out of a total 21 tested (across primary, secondary, and distal outcomes) showed hypothesized intervention-related benefits for this trial of preschool PATHS, the conceptual breadth of demonstrated benefits are notable given that they span several domains of SEC as a multi-dimensional construct. For example, as SEC is viewed by the CASEL (2020) model, the present results have relevance to four out of five CASEL domains. Specifically, results showed beneficial changes in the domain of self-awareness (i.e., identifying emotions) as indexed by children’s emotional knowledge (effect sizes across analyses ranged from 0.22 to 0.23). This type of intervention benefit on emotional knowledge was also evidenced in REDI (Bierman et al., 2008b) and the original preschool PATHS outcome trial by Domitrovich et al. (2007). PATHS related benefits were also demonstrated in the domain of self-management as indexed by one out of three tested aspects of executive functioning, namely working memory (effect sizes 0.36–0.39). This result is conceptually in line with changes in the REDI trial on executive functioning, but the present study results depart from REDI, in that the indicator of EF change/benefits was working memory and not in the realm of inhibitory control. As a whole, these pre to post-test intervention benefits on emotional knowledge and working memory provide partial support for hypothesis 1a (primary outcomes).

The main intervention analyses, also showed partial support for beneficial changes in the domain of social awareness and relationship skills as indexed by positive intervention-related changes in observer-rated prosocial skills across two play occasions. The play task and measurement approach were based on the task as it was used in the REDI trial. This is a particularly robust finding in that ratings were conducted by different research assistants at pre and post-test who observed children in pairs on two standardized play occasions. This finding provides partial support for hypothesis 1b.

In terms of the tested distal outcomes (hypothesis 1c), the present study results are mixed. Notably and unexpectedly, a hypothesis inconsistent intervention-related increase in hyperactivity/impulsivity was found (effect sizes ranged from −0.30 to −0.25). This type of effect for hyperactivity/impulsivity has not been shown in any other preschool PATHS trial in the past. The meaning of this finding should be informed in part by where the means fall on this scale, specifically unadjusted means for PATHS children went from 0.69 at pre-test to 0.86 at post-test, relative to control children means which were 0.63 at pre-test and 0.55 at post-test. This indicates average scores for children in general close to 1 which was Sometimes on a four-point scale (response options are 0 = Never/Rarely, 1 = Sometimes, 2 = Often, and 3 = Very Often). It is possible that this finding is a potential unintended short-term adverse effect of the intervention. However, this finding could also be an artifact of how teachers are rating the children. The control group change in means could be explained by younger children being rated lower than older children, as can happen with behavioral measures of children (as noted by Mihic et al., 2016). As for the intervention group, the change in means could be a reflection of PATHS teachers becoming more attuned to children’s behavior, and maybe also coming to some consensus about standards for child behavior in class as part of their work with PATHS and conversations with other teachers. However, this finding awaits further exploration in other trials and close attention in the long-term follow up of the present study sample. Given the weight of the other positive intervention-related benefits for PATHS in this trial, this unexpected finding while important is outweighed by intervention benefits in emotional knowledge, working memory, prosocial skills.

Moderation analysis by gender indicated a surprising and substantial in magnitude, intervention benefit for emotional knowledge for girls participating in PATHS relative to girls in control schools (effect size was 0.55). Gender moderation of intervention effects was pursued in this study in light of unique intervention benefits evidence for boys in the elementary school PATHS trial within the FAST Track study, on reduced aggression in particular (Bierman et al., 2010). Unlike the PATHS/FAST track trial, all moderated intervention benefits favored PATHS girls with the aforementioned intervention benefit on emotional knowledge as well as reduced anxiety/somatic symptoms as rated by teachers (effect size −0.35). There is great concern about increases in poor mental health among Swedish adolescent girls in general. Thus, the gender-moderated effect for anxiety/somatic symptoms (an indicator of internalizing problems), showing larger intervention-related decreases for PATHS girls relative to girls in the control group, is particularly important in a Swedish public health context.

It is important to reflect on the small group differences on the outcomes tested in this trial. The child tasks and observer-rated play observation tended to show more changes relative to teacher reports, even though some teacher-reported changes were indeed found in this study. It is not typical in Sweden for preschool teachers to rate their students using extensive quantitative assessment batteries. And although we value and find teachers’ views of their students as essential, it could also be that teachers in this trial had less then optimal time to spare due to a heavy workload and thus were not able to invest as much time as they would have liked to complete our rather lengthy surveys, missing was more pronounced on teacher reports relative to child tasks and observations.

Even though there were intervention-related changes in some child tasks, the small group differences in other tasks where effects have been found in prior PATHS trials, could have been affected by sheer number of tasks included in the present assessment battery, with the possibility of increased tiredness in the preschool children across the sessions. Comments from assessors indicated that the Challenging Situations Task (Denham et al., 1994) was sometimes noted as rather difficult for many of the younger children. This task requires that the child can imagine her-/himself in another child’s shoes, and respond to how she/he would feel in a certain conflict situation, and further, give suggestions as to how to resolve the conflict. Many of the same responses were repeated across the different situations presented, and although this may be taken to reflect a consistency in how the child would actually respond to a situation in real life, it could also be seen as an expression of fatigue or difficulty in understanding the hypothetical situations. Regarding measures of EF, the measures of inhibitory control did not show intervention-related effects, although the measure of working memory did. The working memory task may be assumed to have a stronger verbal demand relative to tasks of inhibition, which may partially explain the difference in effect, given the relative emphasis on verbal abilities inherent in the intervention.

### Limitations and Strengths

Although the results of this preschool PATHS trial are for the most part promising with key changes across most of the breath of SEC (in four out of five CASEL domains) and with intervention benefits outweighing any non-effects or one noted adverse effect on hyperactivity/impulsivity, there are several important study limitations to note. Some implementation indicators were missing a substantial amount of information (e.g., teacher-reported lesson coverage missing was at 50%). This is not uncommon in effectiveness trials, indeed in the U.K. trial in the first year of implementation teacher reported lesson coverage was missing at 73%, and was substantially improved in a second year of intervention.

Other limitations are a lack of intervention follow up assessment points. Longer-term follow-up of the children included in this study is needed in order to determine if these short-term effects are maintained over time. This is planned and a follow up study of the PATHS cohort will be conducted in the near future. Findings from comparable studies using the PATHS in the U.S. (e.g., Sasser et al., 2017) suggest that some effects not shown from pre to post-test might be evidenced after some delay, and even minor effects could become more pronounced over time. In the REDI trial’s follow up studies at various time points, for example 5 years following the intervention, children were found to benefit from the intervention in terms of increases in EF over time (Sasser et al., 2017). This was especially so for children with poorer EF at the beginning of the study.

In light of the aforementioned limitations, study strengths included a cluster RCT design, use of multi-method and informant assessment of SEC, namely child task, teacher, and observer report. Several of the assessments used in this trial were also used in two of U.S. PATHS trials (Domitrovich et al., 2007; Bierman et al., 2008a,b) and there was a careful reflection on the cultural adaptation process, as well as training and ongoing support for teachers in this effectiveness trial (Ferrer-Wreder et al., 2020; 2021). This study was a first of its kind, in that it is the first controlled trial of PATHS in Sweden, and in Scandinavia more generally. While these results point to modest generally positive intervention-related benefits (with the exception of the hyperactivity/impulsivity), this trial is important in that it has demonstrated proof-of-concept of the feasibility and potential benefits of conducting such an intervention in an urban Swedish multi-cultural preschool context. Preschool PATHS is a promising intervention for Swedish preschools, and more than a 1-year implementation in schools could yield even more substantial and wide-ranging benefits to children, as was evidenced in the recent U.S. trial of preschool PATHS (see Calhoun et al., 2020).

### General Implications and Future Directions

The preschools in which this study was conducted are part of a wider social welfare (childcare is a right in Sweden) and educational system. Swedish preschools have a national curriculum and high levels of attendance; indeed 85% of eligible children aged 1–5 years attend preschools and as many as 95% of 4–5-year olds (Swedish National Agency for Education: Skolverket, 2020). An aim of the preschool culture and curriculum in Sweden is fostering values and abilities that have much in common with aspects of SEL, such as understanding and showing empathy toward others, and further that school and leisure activities should be guided by democratic principles (Swedish National Agency for Education: Skolverket, 2018). Therefore, effects of an intervention program aimed at strengthening SEL may not be expected to be as large as in settings where this is not already part of a general practice. However, a recent evaluation of preschool quality (Swedish School Inspectorate, SSI: Skolinspektionen, 2017) has identified lack of systematic assessments and routines, lack of shared values, and variation in the quality of the services. Thus, SEL curricula such as PATHS may have promise as an extra complementary tool kit for preschool teachers in their work to support children’s SEL. For more on the Swedish preschool context and culture (see Ferrer-Wreder et al., 2020).

### Practice Specific Implications and Future Directions

A study limitation was that the measurement approach primarily centered on child level outcomes. Other PATHS trials have examined teacher and classroom level effects of PATHS, such as the trial conducted in Turkey (e.g., Seyhan et al., 2019) which found child level benefits as well as positive changes in classroom atmosphere (observer rated), changes in classroom practices by teachers (teacher reported), and improvements in teacher-student relationships as rated by children. Great future potential lies in better understanding how putting a spotlight on social and emotional learning, as is done in PATHS, can benefit and support for positive teaching practices as well as classroom climate. Thus, in terms of practice, it is important to highlight and consider in future research, the importance of teacher practices around social emotional learning and general classroom management as well as overall classroom climate, all efforts to support children in their academic as well as social emotional learning.

Another practice-oriented implication of the present study is the potential that also lies in working to better understand how teachers perceive and can benefit from coaching as part of a larger intervention as well as coaching and ongoing professional development on its own. PATHS is a multi-faceted intervention that includes the actual lessons, puppets, thematically connected children’s literature and extension activities (e.g., crafts, games, teachable moments in everyday life of a classroom). Other essential parts of the intervention support structure for PATHS were teacher training days which brought together preschool teachers and personnel from various schools, a certified PATHS trainer, and research team members. Also, essential to PATHS were visits by PATHS supporters to teachers. The PATHS supporter model involved observation of teachers, feedback and reflection of supporters and teachers, as well as problem solving that was teacher initiated. Other PATHS trials have examined teachers’ perceptions of the intervention support system. In a UK PATHS trial (Ashworth et al., 2018), for instance, 33 teachers took part in a structured qualitative interview about their perception of the coaching that they experienced and across this sample most teachers reported the coaching itself was acceptable and teachers’ particularly valued when coaches provided validation and motivation (Ashworth et al., 2018). Teachers are vital and underutilized co-investigators as well as experts in child development and thus there is also great potential for better integration of participatory research methods with teachers in the social emotional learning field and the interface of this field with teacher education (Schonert-Reichl et al., 2015).

## Conclusion

The World Bank (Sánchez Puerta et al., 2016) and the Organisation for Economic Co-operation and Development (OECD, 2015) have emphasized the value of well-conceived and soundly implemented social emotional learning (SEL) interventions. As stated in a World Bank report “SEL strengthens the healing and coping mechanisms needed to deal with adversity, violence and suffering, essential for healthy development…and contributes to academic success…” (2013, p. 2). Tests of early childhood SEL interventions tend to be predominated by trials conducted in the U.S. If such interventions are to be spread globally and the potential widespread public health impact realized, more empirical examples of rigorously tested and developed SEL interventions in several cultures, which have varied social welfare and educational systems, are needed. This study fits well with this need and the global movement to promote young children’s SEC as a way to protect mental health and ensure improved life chances and opportunities.

## Data Availability Statement

The datasets presented in this article are not readily available because this study’s ethical review does not allow for study data to be in a public repository. For meta-analysis or confirmation of published study results, individual level, de-identified data requests will be reviewed, requests should be made by qualified researchers (e.g., Ph.D.) along with ethical permission under Swedish law regarding secondary data analysis. Requests to access the datasets should be directed to LE, lilianne.eninger@psychology.su.se.

## Ethics Statement

This study was reviewed and approved by the Stockholm Regional Ethics Review Board (dnr. 2012/1714-31/5). Written informed consent to participate in this study was provided by the participants’ legal guardian/next of kin, as well as verbal assent from participating children.

## Author Contributions

LE, LF-W, KE, and TO: manuscript conceptualization and writing—original draft. LF-W, IG, HH, and KE: data curation. KE: formal analysis. LE, LF-W, TO, KE, HH, MA, A-CS, and MS: funding acquisition. LE, LF-W, HH, MA, A-CS, MS, IG, and BH: investigation. LE and LF-W: methodological design. LE, LF-W, and IG: project administration. All authors: writing—review and editing. All authors gave their approval for the publication of this article and its content and agreed to be accountable for attesting to the accuracy of the work represented in this manuscript.

## Conflict of Interest

The authors declare that the research was conducted in the absence of any commercial or financial relationships that could be construed as a potential conflict of interest.
