# Supplementary material for: A Cluster Randomized Trial of Promoting Alternative Thinking Strategies (PATHS®) With Swedish Preschool Children
Source: Front Psychol. 2021 Jul 13;12:695288. doi: 10.3389/fpsyg.2021.695288 (PMC8313762; doi:10.3389/fpsyg.2021.695288)
Supplement: Supplementary file 1 [file Data_Sheet_1.PDF]

ESM1a. Appendix 1a: Flow Diagram, Adapted to Cluster Randomized Controlled Trial (Campbell et al., 2012)

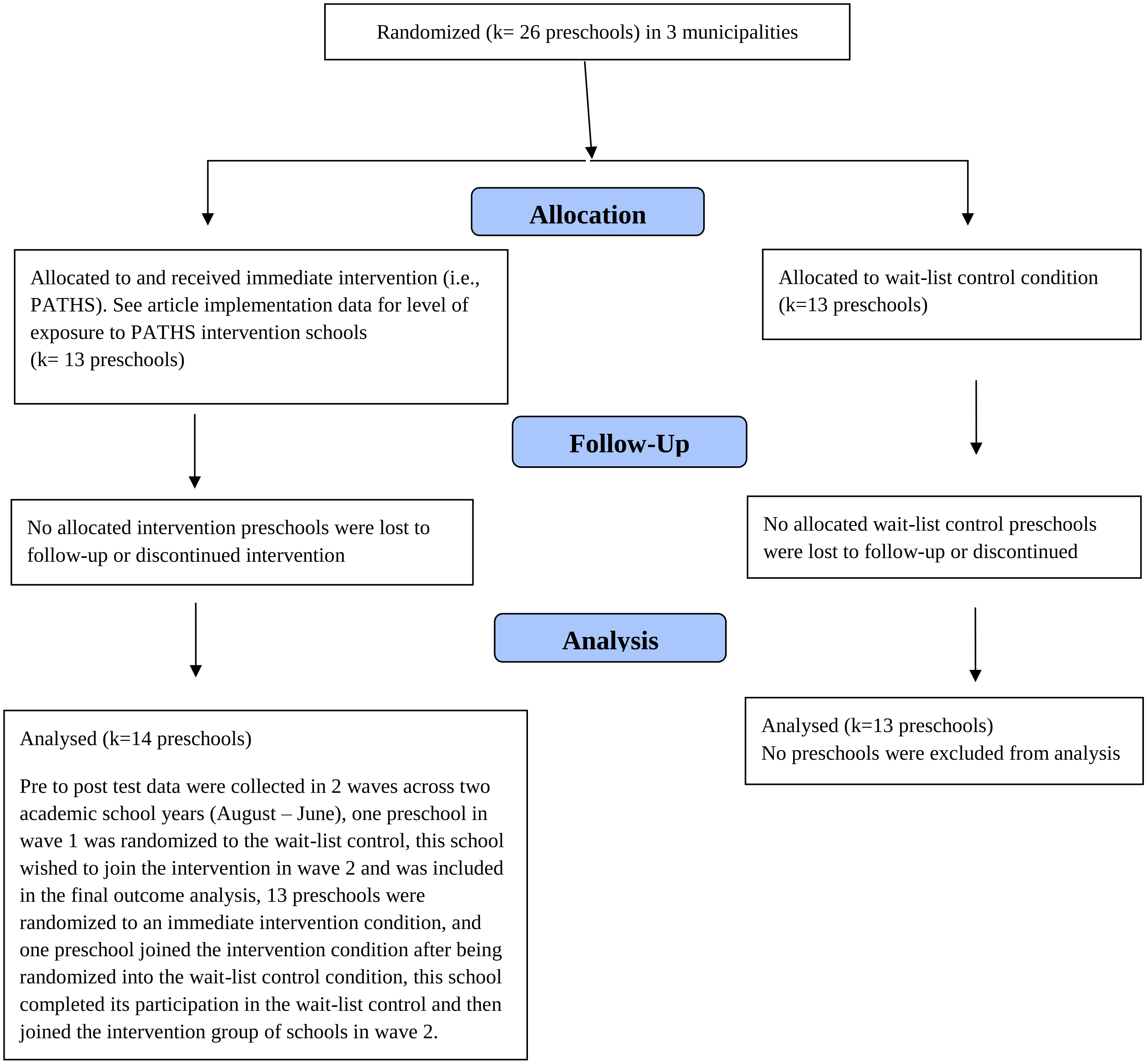

ESM1b. Appendix 1b

Caption to CONSORT 2010 Flow Diagram (Appendix 1a).

Regarding study enrollment, three municipalities that are part of a large urban city were selected by the research team to participate in this study. The rationale behind this selection is described in the method section. Across the selected municipalities, there are on average 50 preschools per municipality that would meet the preschool level study inclusion criteria, the range in number of preschools across municipalities is approximately 33 to 61 preschools. There was a limit to the number of preschools that could be admitted to the study per study wave, due to practical/resource concerns, we recruited schools until the cap or goal number of schools was met in each study wave (2 waves across two years). We aimed to recruit a total of approximately 13 preschools per each study wave, then randomization of schools would follow after the cohort of schools was recruited, in each study wave. Two researchers in the project (LE and LFW) oversaw the randomization sequence and procedure. One researcher placed pieces of paper with the recruited school names written on them into a can that was sealed, shaken well, and then the other researcher pulled out one piece of paper at a time, with the second researcher recording which school was assigned to the intervention or wait-list control condition.

Regarding number and variability in clusters/preschools, in the 13 wait-list control group preschools, there were 22 classrooms that participated in the study. Across these 13 preschools, 54% of schools had one class in one school, 46% had two or more classrooms. The number of classrooms participating in the control schools ranged from one to 4. The number of participating children per classroom ranged from one to 17. In the 14 intervention group preschools, there were 27 classrooms that participated in the study. Across these 14 preschools, 50% of schools had one class in one school, 50% had two or more classrooms. The number of classrooms participating in the intervention schools ranged from one to 5. The number of participating children per classroom ranged from one to 15 children.

Regarding missing data for outcome variables, 17 out of 145 children in the PATHS intervention group have no posttest outcome data (65 have complete data at posttest; 163 have partial data at posttest). For the wait-list control group, 6 out of 140 have no posttest outcome data (77 have complete data at posttest; 157 have partial data at posttest). Figures for the pretest are, for the intervention group, that 5 out of 145 have no pretest data on outcome variables (81 have complete data at pretest; 159 have partial data). For the wait-list control group, 4 out of 140 have no pretest data on outcome variables (62 have complete data at pretest; 174 have partial data). Note that all 285 are included in the analyses because missing data were imputed.

ESM2. Appendix 2: Results in Tables 2 and 3 in detail, all outcomes

Estimated Effects with No Covariates

Primary Outcomes

As shown in Table 2, the difference in the Emotion knowledge score at posttest between the intervention group and control group was estimated to be .31 [-.06, .67], holding constant the pretest score. The effect size, as indexed by the standardized path coefficient was .22 [-.04, .47]. There was a negligible difference between the groups in Emotional Awareness, which was estimated to be .05 [-.24, .32]. For social problem-solving outcomes as measured by the CST, the difference between the intervention and control group at posttest was estimated to be .28 [-.48, 1.03] for Competent responses, -.24 [-.57, .09] for Aggressive responses, and .07 [-.25, .39] for Inept responses. The standardized path coefficients for each of these social problem-solving outcomes was less than +/-.20.

With respect to executive functioning, the difference between the groups in posttest inhibitory control 1 as when measured by the Knock & Tap task was estimated to be .65 [-.71, 2.01], with a standardized coefficient of .12 [-.12, .35]. However, when measured by the Day-Night task (inhibitory control 2), the difference in inhibitory control was estimated to be negative, -2.52 [-5.44, .40], with a standardized estimate of -.20 [-.43, .03]. In contrast, the difference between groups on posttest working memory, as measured by the Word Span task, was estimated to be 1.63 [.49, 2.77], with a standardized coefficient of .36 [.12, .60].

Secondary Outcomes

For teacher-rated prosocial skills outcomes, the difference between the intervention and control group at posttest was estimated to be -.14 [-.42, .13] for Prosocial/Communication Skills, -.14 [-.37, .09] for Emotional Self-regulation Skills, and -.12 [-.42, .18] for Academic Skills. The standardized path coefficients for these outcomes were less than +/-.20. There was a similar pattern for teacher-rated social skills outcomes. Group differences were estimated to be -.11 [-.23, .05] for Social Cooperation, -.09 [-.23, .05] for Social Interaction, and -.06 [-.16, .05] for Social Independence. The standardized path coefficients for these outcomes were also less than +/- .20.

In contrast, the estimated difference between the groups in observer-rated Prosocial/Communication Skills during the play observation was .20 [-.05, .45], with a standardized coefficient of .29 [-.07, .66]. There was a negligible difference in Task Orientation, .02 [-.27, .31].

Distal Outcomes

With respect to internalizing and externalizing problems, estimated differences between the intervention and control groups at posttest were .03 [-.13, .19] for Social Withdrawal, -.07 [-

.22, .09] for Anxiety/Somatic Symptoms, and .14 [-.04, .33] for Aggression. The standardized path coefficients for each of these outcomes was less than +/-.20. In contrast, the estimated group differences for attention problems were positive. For Inattention, this difference was .17 [-.06, .39], with a standardized coefficient of .22 [-.08, .51], whereas this difference was .25 [.06, .44] for Hyperactivity/Impulsivity, with a standardized estimate of .30 [.08, .51].

Estimated Effects with Covariates Included

Primary Outcomes

As shown in Table 3, the difference in the Emotional Knowledge score at posttest between the intervention group and control group was estimated to be .32 [-.01, .65], holding constant the pretest score, age, gender, and community. The standardized path coefficient was .23 [-.01, .46]. There was a negligible difference between the groups in Emotional Awareness, which was estimated to be -.004 [-.39, .39]. For social problem-solving outcomes, the difference between the intervention and control group at posttest was estimated to be .41 [-.24, 1.06] for Competent responses, -.24 [-.55, .07] for Aggressive responses, and .07 [-.20, .35] for Inept responses. The standardized path coefficients for each of these social problem-solving outcomes was less than +/-.20.

With respect to executive functioning, the difference between the groups in posttest inhibitory control 1 when measured by the Knock & Tap task was estimated to be .88 [-.41, 2.18], whereas when measured by the Day-Night task it was estimated to be -1.64 [-5.55, 1.34]. Both standardized estimates were below +/-.20. In contrast, the difference between groups on posttest working memory, as measured by the Word Span task, was estimated to be 1.76 [.63, 2.89], with a standardized coefficient of .39 [.15, .62].

Secondary Outcomes

For teacher-rated prosocial skills outcomes, the difference between the intervention and control group at posttest was estimated to be -.15 [-.41, .11] for Prosocial/Communication Skills, -.15 [-.38, .08] for Emotional Self-regulation Skills, and -.11 [-.42, .19] for Academic Skills. The standardized path coefficients for each of these outcomes was less than +/-.20. There was a similar pattern for teacher-rated social skills outcomes. Group differences were estimated to be -.09 [-.21, .03] for Social Cooperation, -.08 [-.22, .06] for Social Interaction, and -.05 [-.15, .05] for Social Independence. The standardized path coefficients for each of these outcomes was less than +/- .20. In contrast, the estimated difference between the groups in observer-rated Prosocial/Communication Skills as measured in the play observation was .28 [.07, .49], with a standardized coefficient of .41 [.09, .72]. There was a negligible difference in Task Orientation, .07 [-.21, .36].

Distal Outcomes

With respect to internalizing, externalizing, and attention problems, estimated group differences were .04 [-.12, .20] for Social Withdrawal, -.08 [-.23, .07] for Anxiety/Somatic Symptoms, .13 [-.04, .30] for Aggression, and .13 [-.10, .36] for Inattention. The standardized path coefficients for each of these outcomes was less than +/-.20. In contrast, the estimated group difference for Hyperactivity/Impulsivity was .21 [.02, .40], with a standardized estimate of .25 [.02, .47].
